# Visceral Afferent Pathways and Functional Brain Imaging

**DOI:** 10.1100/tsw.2003.93

**Published:** 2003-11-03

**Authors:** Stuart W.G. Derbyshire

**Affiliations:** Department of Anesthesiology, University of Pittsburgh Medical Center, Pittsburgh, PA 15213, USA

**Keywords:** Human, imaging, functional magnetic resonance imaging, fMRI, positron emission tomography, PET, brain, nociception, pain, gastrointestinal, psychosomatic, somatic

## Abstract

The application of functional imaging to study painful sensations has generated considerable interest regarding insight into brain dysfunction that may be responsible for functional pain such as that suffered in patients with irritable bowel syndrome (IBS). This review provides a brief introduction to the development of brain science as it relates to pain processing and a snapshot of recent functional imaging results with somatic and visceral pain. Particular emphasis is placed on current hypotheses regarding dysfunction of the brain-gut axis in IBS patients. There are clear and interpretable differences in brain activation following somatic as compared with visceral noxious sensation. Noxious visceral distension, particularly of the lower gastrointestinal tract, activates regions associated with unpleasant affect and autonomic responses. Noxious somatic sensation, in contrast, activates regions associated with cognition and skeletomotor responses. Differences between IBS patients and control subjects, however, were far less clear and interpretable. While this is in part due to the newness of this field, it also reflects weaknesses inherent within the current understanding of IBS. Future use of functional imaging to examine IBS and other functional disorders will be more likely to succeed by describing clear theoretical and clinical endpoints.

**ABBREVIATIONS**: ACC, anterior cingulate cortex; aMCC, anterior midcingulate cortex; AFP, atypical facial pain; BA, Brodmann area; DC, dorsal column; fMRI, functional magnetic resonance imaging; GI, gastrointestinal; IBS, irritable bowel syndrome; IML, interomedial; MDvc, medial dorsal ventral caudal; MNI, Montreal Neurological Institute; NTS, nucleus tract solitarus; PAG, periaquaductal gray; PET, positron emission tomography; pMCC, posterior midcingulate cortex; rCBF, regional cerebral blood flow; S1, primary sensory cortex; S2, secondary sensory cortex; SPM, statistical parametric mapping; VMpo, ventromedial posterior; VPL, ventral posterior lateral; VPI, ventral posterior inferior

## SPECIFICITY AND PATTERN THEORIES OF PAIN

Almost 350 years ago, Descartes[1] proposed that a noxious stimulus transfers energy to "threads" running through the body to open pores in the brain and signal pain. Pain was thus produced just as pulling on a rope might cause a bell to ring. Descartes was proposing a theory of pain based on the specificity of the stimulus and a dedicated processing system designed to receive specific stimulus information. In the modern interpretation, pain fibers, rather than threads, are stimulated and relay information to a hypothesized pain center somewhere in the brain, as illustrated in Fig. 1.

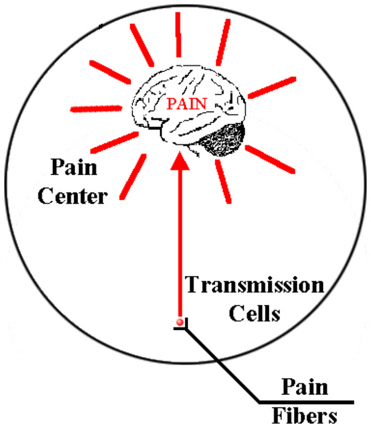

**FIGURE 1**. Specificity theory. Peripheral nerve fibers deliver impulses to transmission cells that relay information to a proposed pain center in the brain.

During the past century, special effort was made to place the pain center in primary sensory cortex (S1) because of the known association of S1 with sensorial experience. Experimental attempts to demonstrate S1 as the pain center, however, were largely unsuccessful. Penfield[2], for example, stimulated 800 areas of somatosensory cortex but found only 11 to elicit any sensation of pain. Lesions of somatosensory regions have been attempted to treat phantom limb pain but without success[3]. Phantom limb pain is itself a challenge to specificity theory because of the missing relationship between injury and pain.

The limitations of specificity theory led to the development of alternative theories of pain including pattern theories that suggest pain only in the presence of an array or summation of active fibers and neurons[4]. Pattern theories help to explain why a receptor can be physiologically specialized to receive certain sensations, but nevertheless fail to generate a consistent psychological experience. A stimulus might fail to capture experience, or may generate an unexpected experience, dependent on other processing activities as has been demonstrated, for example, with the thermal grill[5]. The thermal grill consists of warm (40ºC) and cool (20ºC) metal bars, which are not independently noxious. When the bars are interleaved, however, the resulting sensation is a burning pain similar to a cold burn. Fig. 2 illustrates the dual pathway system that is thought to give rise to the painful illusion. One pathway codes for cool and noxious cold temperatures and projects to the thalamic ventromedial nucleus (VMpo), while a second pathway through the ventral caudal part of the medial dorsal thalamus (MDvc) codes for noxious cold, heat, and pinch. Whereas the cool bars activate both pathways, the warm bars only inhibit the cool pathway resulting in a net transmission through the MDvc and an experience of burning cold. Thus a stimulus that is inherently non-noxious and not a threat to tissue nevertheless generates an experience of pain.

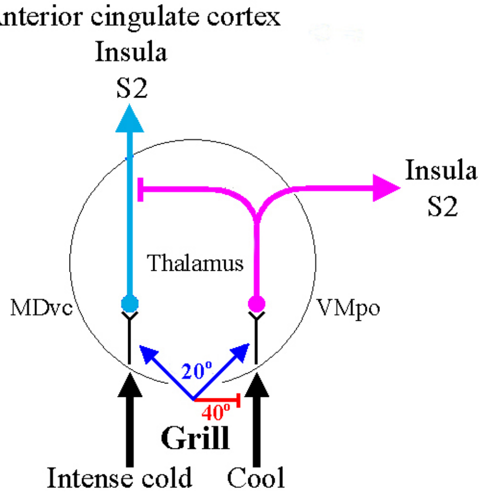

**FIGURE 2**. A schematic representation of how the thermal grill might produce an illusion of pain and illustrates the pathways described by Craig[5] Cool stimuli excite the spinothalamic pathway through the VMpo (purple), which subsequently inhibits the pathway through the MDvc (turquoise). In the thermal grill experiment, both pathways are excited by the 20ºC stimulation (blue). The warm, 40ºC, bars (red), however, also inhibit the VMpo, but not the MDvc pathway, resulting in less excitation entering the inhibitory pathway. The net output is through the MDvc pathway to the anterior cingulate cortex resulting in the experience of a burning pain. Developed from Derbyshire[8].

This explanation of the thermal grill illusion is still dependent on hypothesized dedicated line systems for sensations, which remains controversial[6,7]. A functionally and anatomically well-organized response to noxious stimulation does not sit comfortably with the large number of brain areas involved in pain processing and other sensory experience[8,9]. There remains an important "explanatory gap" with regards to our understanding of the connection between distinct mental states, such as "blueness" or "pain", and the associated neural correlates[8,9,10,11]. Nevertheless, notions of sensory processing built on hypothetical patterns of unspecified connections between distant areas of the nervous system have not provided researchers and clinicians with useful ways to approach scientific questions[7,12].

Another attempt to reconcile the differences between the opposing concepts of specificity and pattern generation is that of Cervero and Janig[13] to explain visceral pain. According to their model, spinal cells receive convergent input from low-threshold mechanorecptors and high-threshold nociceptors present in the viscera. Low-threshold information is received by wide-dynamic range spinal neurons, which mediate nonpainful sensations and reflex regulatory responses. These fibers are suggested to also project to neurons that process noxious stimuli, but at subliminal levels only. Transient, high-intensity stimuli activate high-threshold spinal neurons that activate the ascending nociceptive pathway triggering acute visceral pain. Low-threshold fibers, however, are also activated by high-intensity stimuli and, over time, it is suggested that mere low-threshold input becomes sufficient to generate painful experience. Previously unresponsive "silent nociceptors" are proposed to maintain pain even in the absence of an afferent barrage during some chronic visceral disorders. Normal regulation of the organ may now be sufficient to activate sensitized neurons resulting in disorders such as the irritable bowel syndrome (IBS)[14]. These mechanisms are illustrated in Fig. 3.

Interestingly, Kern et al.[15] have recently demonstrated activation of S1 during subliminal rectal distension and further activation of anterior cingulate cortex (ACC) and insula in female subjects only. These latter regions are thought to play a special role in visceral sensation and may relate to IBS disorder, which has a female dominance[16,17].

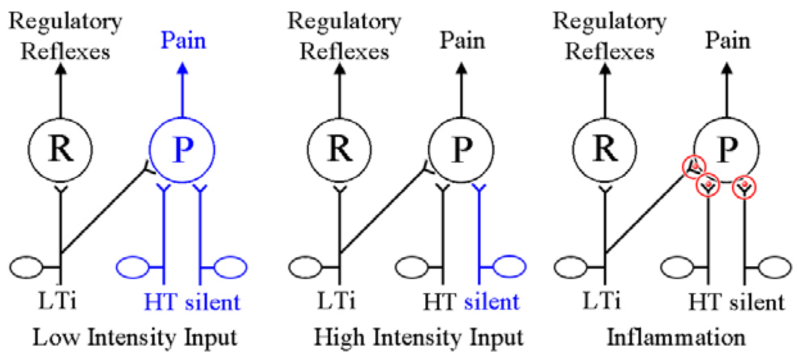

**FIGURE 3**. A schematic representation of the hypothesized roles for different visceral receptors. Visceral afferents are shown to activate two central mechanisms, one responsible for regulatory and nonpainful responses (R), and the other for pain experience (P). Normal regulatory activity is shown to the left. Low-threshold input is insufficient to activate the pain mechanism, which is shown as deactivated (blue). Transient, high-intensity input activates both the low- and high-threshold afferents triggering pain sensation. Prolonged stimulation, such as inflammation, also activates the so-called silent nociceptors and sensitizes the central inputs of both high- and low-threshold afferents (shown in red). Adapted from Cervero and Janig[13].

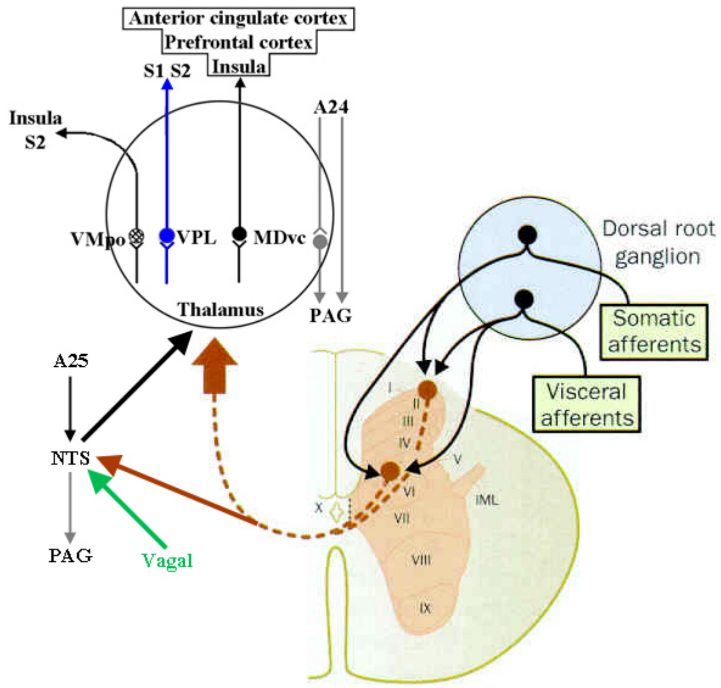

**FIGURE 4**. Viscerosomatic convergence of primary afferent fibers on neurons of lamina I and V of the spinal dorsal horn. Supraspinal and vagal projections to the NTS and thalamus are shown with subsequent projection to cortical structures and descending projection to the PAG. Adapted from Janig[14], Cervero[12], and Derbyshire[18]. See text for further details. IML = interomedial cell column; MDvc = ventral caudal part of the medial dorsal nucleus; VPL = ventral posterior lateral nucleus; VMpo = posterior part of the ventral medial nucleus; S1 = primary somatosensory cortex; S2 = secondary somatosensory cortex; PAG = periaquaductal gray; NTS = nucleus tract solitarus.

## CENTRAL TRANSMISSION

The traditional view of the central transmission of visceral pain is that convergent viscerosomatic neurons project onto crossed anterolateral pathways, mainly the spinothalamic and spinoreticular tracts, which carry the afferent signals into the brain. The crossover and ascending system is illustrated in Fig. 4.

These ascending tracts from the periphery and viscera arrive at multiple terminations within the thalamus before being transmitted to higher cortical centers. These transmissions are often divided according to the medial and lateral pain systems[8,19,20]. Definition of the lateral and medial pain systems depends on the divergence of spinothalamic projections in the thalamus. In terms of the lateral pain system, spinothalamic projections are largely to the VPL and VPI (ventral posterior inferior) nuclei. These nuclei project in turn to primary (S1) and secondary (S2) somatosensory cortices as shown in Fig. 4.

These pathways transmit information regarding the intensity, duration, and location of noxious stimuli with few alterations in coding parameters between the spinal cord and cortex and are thus ideal for providing information about the location and characteristics of a noxious stimulus. The poor localization of visceral sensation suggests that this pathway would not be strongly activated by visceral stimulation. It is shown in blue in Fig. 4 to illustrate this potentially "silent" response.

The medial pain system comprises spinothalamic projections to medial thalamic nuclei and from there to limbic cortices including the ACC. In addition, the PAG has reciprocal connections with the medial thalamic nuclei and receives a direct input from the ACC. Neural terminations arising from medial thalamic nuclei are ideal for providing motivational and affective qualities associated with noxious stimulation, which should be activated by visceral stimuli that have a dominant affective component.

Fig. 4 shows an additional projection from VMpo to the insula and S2, described by Craig et al.[21] as a specific pathway for heat pain information. The VMpo region is, however, also contiguous with ventromedial thalamic projections that receive vagal visceral input and project onto the insula[22]. Thus, somatic sensory input and visceral information converge in the insula where sensory and emotive feelings may be integrated.

More recently, however, the dorsal column (DC) pathway has been discovered to carry visceral information[23,24,25]. The DC system has traditionally been viewed as a pathway responsible for the discriminative aspects of tactile sensations and for kinesthesia. A DC lesion in monkeys has been demonstrated to reduce the response of VPL neurons previously excited by colorectal distension. Severing the spinothalamic tract, in contrast, has failed to reduce VPL responses to noxious distension. Unfortunately, no recordings were taken from the medial group of thalamic nuclei.

These findings raise the possibility of a separate visceral pathway dedicated to the transmission of at least noxious colorectal distension and possibly other types of visceral pain. As for somatic pain, there has been renewed debate as to whether such ideas are consistent with pattern theories of pain or are more synonymous with the concept of specificity theory[12]. Clearly, whether visceral or somatic, the sensation of pain is complex involving multiple psychological components, ascending pathways, and receptor systems suggesting that many areas of the central nervous system will be involved in processing noxious information. Variability in the regions and patterns of activation is to be expected when using different stimulus techniques. To the extent that one stimulus evokes different psychological experience, involves alternate pathways, or receptor systems to another, however, it can be predicted that the consequent neural processing will be altered in predictable and consistent fashion. If visceral and somatic processing involve different systems and experiences, therefore, this should be apparent in the central circuits that can now be revealed using powerful functional imaging techniques.

## BRAIN IMAGING

Functional imaging techniques now exist that can be used to directly investigate brain responses to noxious stimulation. The cerebral networks that participate in somatic pain perception have been extensively studied using positron emission tomography (PET)[26,27,28,29,30,31,32,33,34,35,36,37,38,39,40,41,42,43,44,45,46,47]. Studies have also been performed using visceral sensation[48,49,50,51]. PET scanning utilizes biological molecules synthesized with radiolabeled isotopes, such as $^{15}$O-labeled water to track cerebral blood flow (CBF) and thus monitors regional changes in this parameter (rCBF). In the normal brain, rCBF is closely coupled to regional cellular metabolism, therefore $^{15}$O-labeled water serves as a reliable monitor of cerebral interneuronal activity[52]. Alterations in rCBF can then be mapped to specific intracerebral structures and foci, using either magnetic resonance imaging (MRI) or a computerized stereotactical method[53,54].

Functional magnetic resonance imaging (fMRI) has also been applied to the study of somatic[55,56,57,58,59,60,61] and visceral[15,62,63,64,65,66,67,68,69] pain processing. The physical basis of fMRI is the difference in the magnetic properties of oxygenated and deoxygenated blood, specifically the difference in decay time of the MR signal from oxyhemoglobin and deoxyhemoglobin. This difference in signal characteristics allows for the localized detection of rCBF similar to that from PET imaging but with potentially greater temporal and spatial sensitivity. In overview, the process is as follows: The subject is placed into a strong and homogenous magnetic field where the various atomic nuclei, particularly the proton nucleus of the hydrogen atom, align themselves to “magnetize” the individual. The proton nuclei precess about the applied field at a characteristic frequency but at random phase to one another. The result is that the precess of each proton sends out an MR signal that is lost among the signals of the many other protons. Application of a brief radio frequency electromagnetic pulse introduces a transient phase, coherent to the applied magnetic field strength, such that the precess now returns a radio signal that can be detected and formed into an image. The signal decay rate of deoxyhemoglobin is more rapid than that of its oxygenated counterpart allowing for the differential detection of the increase in oxygenated blood as rCBF during activation studies.

A large number of imaging studies have now been performed using either a phasic (intermittent) or tonic (continuous, no fluctuations) somatic pain stimulus (see Derbyshire[8,9,18] for reviews). A smaller number of studies have used visceral distension of either the esophagus or colon (see Derbyshire[70] for review). To provide an illustration of the demonstrated central response, grids corresponding to the Talairach and Tournoux atlas[71] were fitted by eye to the standard MNI brain provided with SPM99. The grids were aligned to relevant anatomical landmarks on the lateral and medial surface of the brain. Each reported activation was then color coded and plotted onto the surface using the grid as a guide. Wherever possible, the positioning of each reported activation was checked against the primary reported figures and was further ensured to be within or adjacent to the Brodmann area (BA) reported by the primary authors.

Finally, Brodmann’s regions were placed on the lateral and medial surface according to those reported by Talairach and Tournoux[71] but also according to the reports by Vogt et al.[46,72] and Derbyshire[9] for the medial surface. The division of the ACC into midcingulate (aMCC and pMCC) and perigenual cingulate (A24) is motivated by the different connections of these regions. These differences are described in detail elsewhere[72,73,74] In summary, perigenual cingulate receives most mediodorsal thalamic and amygdala afferents[75,76], whereas midcingulate has major and reciprocal connections with cingulate premotor areas, the pontine nuclei, and area 46

of the prefrontal cortex[73]. Thus the midcingulate region is liable to be involved in executive motor function and decisions including response selection during divided attention tasks[77,78], while perigenual cingulate is more likely to be associated with affective responses[79,80,81].

The further subdivision of the midcingulate region into aMCC and pMCC is motivated by the observation that the amygdala projections into the perigenual cingulate encroach onto the anterior subsection of midcingulate, denoted aMCC[82]. In addition, aMCC has a relatively higher density of opioid receptors than pMCC, similar to perigenual cingulate[83]. There is also an intermix of activation relating to cognitive and affective processing in aMCC apparent from a recent meta-analysis of studies[84]. Finally, aMCC and not pMCC is the region demonstrated by Rainville et al.[41] to activate in proportion to the degree of experienced unpleasantness and was exclusively activated during pain experience compared to performing the Stroop task[78]. aMCC conforms to the criteria of Vogt[74] as a region liable to be involved in processing the affective component of pain. In addition, Vogt and Derbyshire[85] have recently described cytoarchitectural differences between aMCC and pMCC further supporting this subdivision.

## RELATIVE NEURONAL RESPONSES

Fig. 5, 6, and 7 show a complex and potentially confusing variety of neuronal activations during somatic and visceral sensation. Fig. 5 illustrates the responses on the medial surface and demonstrates that both upper and lower gastrointestinal (GI) distension activate midcingulate including both pMCC and aMCC. Phasic and tonic somatic pain also involves activation of these regions. The small number of visceral studies makes it currently difficult to decide if the extensive spread of activation to include BA 23 and the motor cortices (BA 6, 8, and 4) is more unique to somatic pain. Certainly on the right hemisphere there is very little apparent difference in the pattern of activation within this region when comparing somatic and visceral responses.

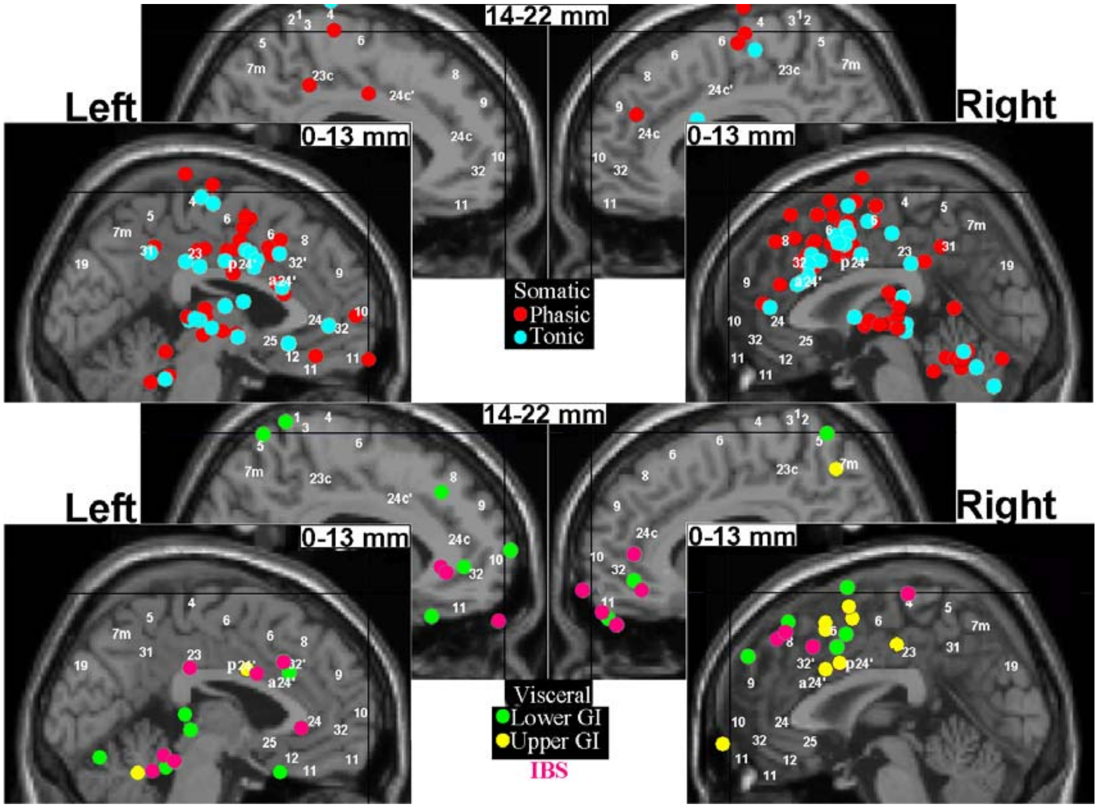

**FIGURE 5.** The center of each reported rCBF increase on the medial surface of the brain during the experience of phasic or tonic somatic pain (top) and during the experience of visceral pain (bottom) in the lower or upper gastrointestinal (GI) tract. Responses to lower GI distension in IBS patients are shown in purple. The responses are plotted onto the standard MNI brain provided with SPM99 and the Brodmann's areas shown based on translation from the atlas of Talairach and Tournoux[71], Vogt et al.[46,72], and Derbyshire[9]. The ACC is divided into perigenual (24), anterior midcingulate (a24') and posterior midcingulate (p24') as explained in the text.

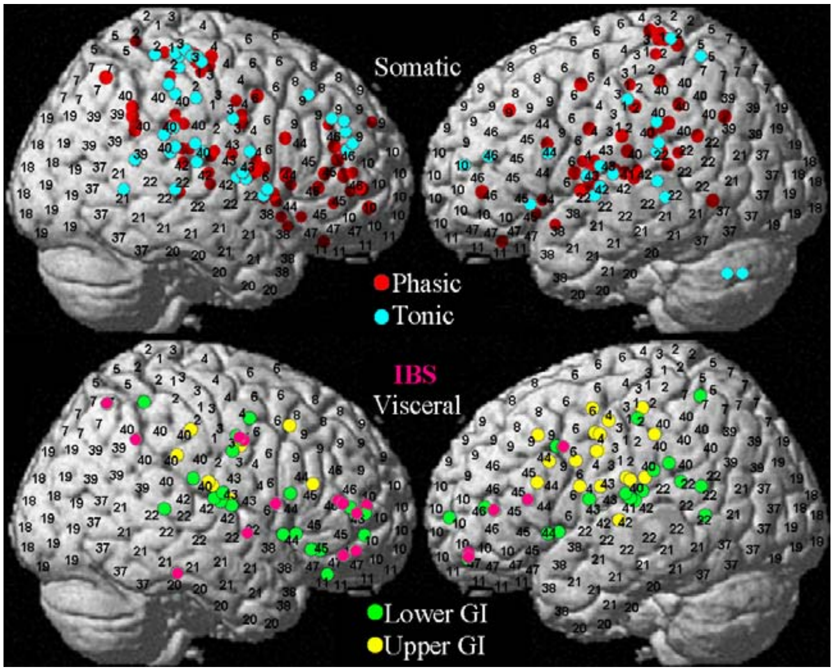

**FIGURE 6.** The center of each reported rCBF increase as for Fig. 5 but plotted onto the right and left lateral surface.

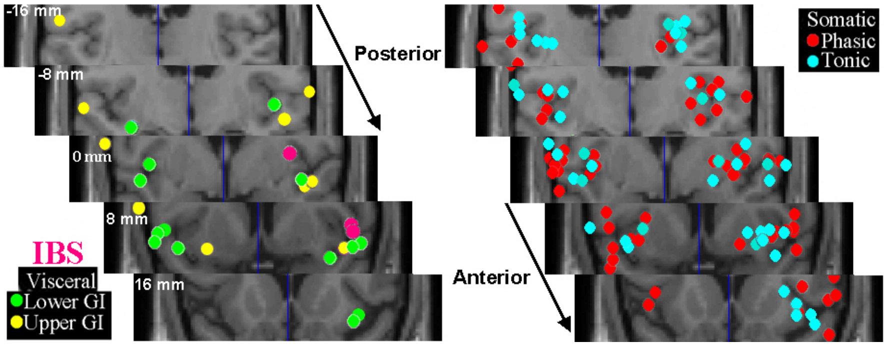

**FIGURE 7.** The center of each activation locus along the posterior-anterior extent of the insula. The color coding is shown in the key box and is as for Figs. 5 and 6. The arrows show the direction from posterior to anterior for the two posterior (behind the anterior commisure) and anterior (in front of the anterior commisure) slices as labeled. The most posterior section shows the responses –16 mm behind the anterior commisure, the most anterior section 16 mm anterior to the anterior commisure.

Within the perigenual and more ventral regions of the ACC, however, there is clearly a greater visceral response specific to stimulation of the lower GI tract. In addition, a cluster of activation in the left hemisphere can be observed around the posterior edge of the brain stem during lower GI distension. This brain stem region may include the NTS, although there may also be a representation of the midbrain PAG. The resolution of the various imaging techniques adopted is currently insufficient to distinguish these relatively small and adjacent regions, but the concentration of many studies in the same location provides increasing confidence that common brain stem structures are being engaged.

The NTS has extensive direct monosynaptic bidirectional connections with both the hypothalamus and the amygdala and with the parabrachial nucleus and the PAG[86]. The perigenual and ventral anterior cingulate cortex (A24 and A25) receive substantial direct input from the hypothalamus and amygdala while A25 has direct monosynaptic input to the NTS and A24 to the PAG[82]. This circuitry represents a system capable of mediating important autonomic visceral response, affective integration of visceral events, and descending modulation of viscerosensory traffic. The lack of any reported activation within this circuitry during esophageal stimulation is suggestive of differential visceral control functions during rectal and esophageal stimuli. This suggestion needs to be confirmed via independent study, but may have implications for the understanding of upper and lower functional GI disorder.

A very notable difference between somatic and visceral stimulation can be observed in the thalamus. Somatic stimulation provides extensive bilateral activation of the thalamus that is almost completely absent during visceral stimulation. Nevertheless, the apparent lack of thalamic response from the studies of visceral distension is surprising, as information arising from the viscera that reaches conscious perception would be expected to synapse in the thalamus before reaching primary sensory or motor regions. Primary motor and sensory cortices can be observed in Fig. 6 during studies of visceral sensation, suggesting that the lack of thalamic report may be a false negative. Some visceral studies have reported thalamic activation, but did not include coordinates to allow a plot on Fig. 5[67,68]. Furthermore, thalamic activation has been described in animals during both colorectal and esophageal distension[23,24,25]. It seems likely that visceral sensations do correlate with some thalamic activation, but this may be less than observed during somatic experience. Further studies will be necessary to clarify the nature of any thalamic differences in processing.

The amygdala inputs into area 24 have been noted as substantial and reciprocal but the cortical regions receiving the heaviest amygdaloid afferents, with much reduced reciprocal projections, are the medial and lateral orbitofrontal cortex, medial wall of the frontal cortex, and the anterior insula[82,87]. Medial and lateral (see Fig. 6) orbitofrontal cortices are also differentially represented during rectal as opposed to esophageal distension and further suggest the possibility of altered autonomic response and control during lower as opposed to upper GI stimulation. Differences between somatic and visceral stimulation, however, are perhaps less apparent on the lateral surface than might be expected. Both activate S1, though possibly less with visceral stimulation and in clearly different subregions, S2 and prefrontal cortex. These latter activations show a tendency to be more bilateral and inferior during lower GI than somatic stimulation, but there are too little data to be more definitive. At least, the common activation of S1 and S2 suggest that there is a shared viscerosomatic pathway to these cortical structures, as well as to the midcingulate sites described previously.

The response of S2 during stimulation of upper and lower divisions of the GI tract is extensive and supports previous suggestions that the predominant sensory representation of visceral stimulation occurs in S2[88,89]. S2 has a coarse somatotopic representation and may also have some viscerosomatic organization, but this has not been demonstrated and there is no obvious separation of upper and lower GI sensation in S2 apparent from Fig. 6.

There is a cluster of reported activation around the intra-abdominal region of S1 on the right hemisphere during GI distension and on the left hemisphere there is activation of a more superior portion of S1, possibly corresponding to the trunk region. The relatively dense spinal innervations of the peri-anal and proximal divisions of the GI tract suggest these latter activations may be dependent on spinothalamic transmission directly, or indirectly via the ventrolateral medulla or midbrain PAG region, into the thalamus[88,89]. Alternatively, or perhaps additionally, there is a

DC projection to the brain-stem gracile nucleus that projects to the VPL thalamic nuclei as recently described[23]. Somatic S1 activations are more extensive and mostly tightly clustered around the hand and arm region of S1 reflecting the common use of the hand and arm during somatic studies.

Insula activation is evident during both upper and lower GI stimulation and during somatic pain. Activation of insula is the most consistent finding during both noxious visceral and somatic sensation[8,9,70]. The somatic responses are notably spread throughout the insula, whereas the visceral responses concentrate in the midanterior section, which is associated with internally generated emotion and has been designated as a visceral sensory area by Neafsey et al.[90] Electrical stimulation of the insula in rats, cats, dogs, monkeys, and humans elicits changes in blood pressure, heart rate, respiration, piloerection, pupillary dilatation, gastric motility, peristaltic activity, salivation, and adrenaline secretion[90].

There are multiple routes to the insula including a direct termination from the recently described ventromedial posterior nucleus in the thalamus and terminations from parabrachial nucleus, the lateral hypothalamic area, the central nucleus of the amygdala, and cingulate and orbitofrontal cortices[22,90]. Craig et al.[22] have recently described the VMpo projection to insula as contiguous with ventromedial thalamic projections that receive vagal visceral input. Thus, somatic sensory input and visceral information converge in the insula where sensory and emotive feelings may be integrated. From the evidence presented, esophageal sensation has a visceral representation limited to this convergent region whereas rectal sensations extend into other visceral and autonomic control centers.

Unlike the anterior insula, the posterior region has predominantly sensory and motor connections that include S1 and S2 and premotor area 6, which in turn provides the principal cortical input into M1[91]. In addition, whereas the anterior section of the insula receives projections from perigenual cingulate, the more posterior sections receive pronounced input from midcingulate[82]. Although both visceral and somatic stimulation have resulted in midcingulate activation, there is a distinct trend towards greater perigenual cingulate during lower GI sensation and less response during somatic pain and no response during upper GI stimulation. The midcingulate region has been suggested as predominantly controlling response selection and has particularly heavy connections with the premotor cortex (medial area 6), which also shows potentially greater activation during somatic pain.

Overall, the pattern of response during esophageal distension might be described as having both somatic and visceral features. One interpretation is that the presented neural activation for upper GI sensation is ideally suited to the parallel processing of skeletomotor and autonomic motor responses. In contrast, lower GI sensation involves largely limbic, autonomic, and affect associated circuits.

Finally, there is a very apparent difference in activity in the prefrontal cortex with lower GI stimulation resulting in the activation of more anterior prefrontal regions (in and around BA 10/46) and upper GI stimulation producing less overall frontal activity and in more posterior and superior prefrontal regions (in and around BA 9/44). The prefrontal activity during lower GI stimulation concentrates in regions that may be described as more “heteromodal”[91]. BA 10/45/47/11 receive widespread neural inputs from unimodal areas in all the major sensory modalities, from all other heteromodal regions of the brain, and from many limbic areas[91]. These prefrontal neuronal responses can be specialized, mixed, or multimodal and can integrate vast amounts of information to provide control outputs for varied cognitive and motor operations. The role of prefrontal area 10, for example, has been described as mediating a function of holding in mind primary goals while exploring secondary possibilities as part of the process of planning and reasoning[92].

Disappointingly, the differences between IBS patients and control subjects were minimal and without obvious meaning. Control responses in the primary and secondary somatosensory cortices appear greater than those of IBS patients that may be concordant with reports of somatic hyposensitivity in IBS patients[93]. A more recent report, however, has demonstrated hypersensitivity to somatic stimulation in IBS patients[94]. Future studies may attempt to relate somatic sensitivity in IBS patients to activation within S1 and S2 to provide some further insight regarding these conflicting reports. The finding of a different pattern of activation between IBS and control subjects within sensory regions is not easily integrated with current clinical understanding of IBS as discussed in the next section.

## CLINICAL IMPLICATIONS

The dominant themes in the clinical understanding or interpretation of functional GI disorders include hypersensitivity, hypervigilance, hyperalgesia, central sensitization, stress mediated dysfunction, and, encompassing all these themes, the biopsychosocial model of visceral disorder[95,96,97,98,99]. These themes have their analogy in the discussion of some somatic pain disorders including atypical facial pain (AFP) and other “functional disorders”[32,100].

The suggestion of hypersensitivity follows the observation of patients as being more sensitive than control subjects when examined using balloon distension of the esophagus, stomach, small intestine, bile duct, and colorectum. Numerous trials with IBS patients have provided evidence of reduced pain and discomfort threshold during rectal distension compared with controls that may be further increased following rapid repetitive stimulation of the sigmoid colon[97]. Somewhat analogously, repetitive distension of the esophagus has been shown to reduce pain threshold in patients with functional dyspepsia but not controls[101].

This hypersensitivity can occur as a consequence of inflammation or infection with secondary hyperalgesia and allodynia following sensitization of peripheral nociceptors and excitability of spinal dorsal horn neurons. This phenomenon has been demonstrated experimentally in patients with noncardiac chest pain[98]. The role of higher central, psychological involvement is inferred from studies demonstrating development of IBS following a bout of gastroenteritis only in patients with high levels of life stress and hypochondriasis[102]. The suggestion that functional GI patients may be hypervigilant towards their organ of distress and symptoms is supported by studies demonstrating selective recall and biased information processing in IBS patients[103,104]. The biopsychosocial model of functional GI illness encapsulates the multimodality inputs and processes that can precede and maintain visceral dysfunction and distress[105].

The results of functional imaging studies can be brought to bear in support of these concepts. It is evident that visceral sensation produces activation in large and widespread regions of the brain that integrate stress, cognition, and visceral sensory information. Disruption in the processing of these regions may explain some of the specific symptoms of the disorder. In general, differences in activation of perigenual cingulate, midcingulate, orbito- and prefrontal cortices in patients with visceral or somatic dysfunction might be expected to reflect changes in autonomic response, stress experience, and cognitive processing during stimulation or normal regulatory function. Activation of prefrontal area 10 and perigenual cingulate cortex (A24) during the simple expectation of rectal distension in IBS patients, and the exaggerated cingulate response to esophageal distension in subjects primed with a negative affective context, provide evidence that these circuits can be modulated independent of GI stimulation[50,51,89]. Enhanced ACC processing of acute pain stimuli following hypnosis to increase the affective experience of pain and in patients with AFP further suggests that these circuits underlie important aspects of functional disorder and can be adapted according to changes in psychological processing[32,41]. The findings of Kern et al.[15] and Berkley[16] suggest the real possibility that subliminal distension can activate the ACC in female volunteers, and the ACC may process sensation abnormally in IBS and AFP subjects, which have a large female dominance.

Nevertheless, the flow of information from functional imaging studies of visceral sensation is yet to provide closure on the nature of any specific process involved in the dysfunction of the GI

or somatic system. This is not entirely a problem of functional imaging. The concepts adopted to interpret functional disorder are themselves broad and their relevance and utility has recently been questioned[106,107]. The prevalence of rectal hypersensitivity in IBS, for example, is highly variable across studies ranging from 20–80%; discovery of hypersensitivity is usually dependent on repetitive stimulation and has only a weak correlation with current pain ratings. Rectal hypersensitivity does not predict therapy outcome and has yet to contribute to the diagnosis or pharmacotherapy of IBS[106]. Recent evidence has also called into question the specificity of visceral sensitivity to the lower GI tract and the existence of somatic hyposensitivity in IBS disorder[94,108]. The gender difference in somatic pain populations also appears to be only loosely related to differences in pain threshold or tolerance, which are also highly variable[16,109]. Equally, the results from functional imaging studies have been inconsistent with studies of both somatic and visceral pain disorders revealing less[51], more[32,67], and the same[35] ACC response as control subjects. Gender differences in somatic and visceral pain responses are inconsistent with greater insula response reported for males[49,110] and females[15,39] during visceral[15,49] and somatic stimulation[39,110].

The technique of functional imaging to investigate these currently uncertain disease markers and the use of a broad interpretative framework such as the biopsychosocial model can lead towards a line of reasoning that does not necessarily advance understanding. Interpretation of a set of activations using the biopsychosocial model of disease relies on a large theoretical construct itself dependent on multiple components not directly addressed by the study. The result is a set of activations that can be easily integrated into current conceptual thinking while remaining indeterminate as sources of evidential relevance for any component of the disease experience[111]. To state that differently, providing evidence that is only “consistent with” general theoretical claims risks leaving the speculative nature of those claims undisturbed by the passage of experimentation[11]. Theories require explanations of processes and, at least for now, that is critically absent. Although further exploratory work will add to the overall picture and understanding of central response to visceral sensation, a focus on hypothesized regional responses to psychophysically well controlled stimuli is more likely to push the field forward.

## ACKNOWLEDGEMENTS

The author is supported by a grant from the Pittsburgh Foundation and the John F. and Nancy A. Emmerling Fund. This article is dedicated to the memory of Manuel J. Manolios.
